# The *Brucella* effector protein BtpB facilitates STAT3 activation to regulate polarization in macrophages during infection

**DOI:** 10.1186/s13567-026-01833-8

**Published:** 2026-09-02

**Authors:** Ye Yuan, Minghui Wang, Mingyue Hao, Jialu Chen, Xiaofang Liu, Yuanhao Yang, Yimeng Cui, Shengnan Li, Wei Liu, Dong Zhou, Wei Liu, Yaping Jin, Aihua Wang

**Affiliations:** 1 College of Veterinary Medicine, Northwest A&F University, Yangling, 712100 China; 2 Key Laboratory of Animal Biotechnology of the Ministry of Agriculture, Northwest A&F University, Yangling, 712100 China; 3 Engineering Research Center of Efficient New Vaccines for Animals, Ministry of Education, Yangling, 712100 China

**Keywords:** *Brucella*, T4SS, BtpB, macrophages, M1 polarization, STAT3

## Abstract

*Brucella* is a Gram-negative facultative intracellular pathogen that causes widespread zoonotic infections. Macrophages are crucial immune and antigen-presenting cells that differentiate into proinflammatory M1-type or anti-inflammatory M2-type cells in different microenvironments. The BtpB protein of *Brucella* is a type IV secretion system effector protein that modulates host inflammatory responses by inhibiting Toll-like receptor signaling and controlling dendritic cell activation. A high expression of intracellular proinflammatory factors is induced in *B. suis* deficient in BtpB (*B. suis* mutant strain Δ*btpB*). However, the role of BtpB in macrophage polarization triggered by *Brucella* infection is uncertain. In this study, RAW264.7 macrophages were utilized as a model to investigate the impact of BtpB on macrophage polarization. The cells were infected with wild-type *B. suis* strain S2, *B. suis* mutant strain Δ*btpB*, or the *B. suis* complemented strain C-Δ*btpB* followed by flow cytometry, reverse transcription quantitative PCR (RT-qPCR), western blotting, immunohistochemistry, and metabolic detection analyses. The expression of BtpB suppressed M1 polarization and promoted M2 macrophage polarization. Moreover, BtpB upregulated the expression of signal transducer and activator of transcription 3 (STAT3) in host cells. Inhibition of STAT3 promoted the expression of nitric oxide synthase (NOS2) in cells infected with either *B. suis* or C-Δ*btpB*, with no observable differences compared to cells infected with Δ*btpB*. Conversely, STAT3 overexpression resulted in a downregulation of NOS2 levels in both wild-type and mutant cells. These findings were corroborated by messenger RNA (mRNA) assay and enzyme-linked immunosorbent assay (ELISA) that confirmed the regulatory role of STAT3 in modulating NOS2 expression. Additionally, intracellular proliferation assays under STAT3-modulated conditions indicated that STAT3 suppresses replication of *Brucella*. In conclusion, the results demonstrate that BtpB inhibits M1 polarization in macrophages by regulating STAT3 expression which provides a strong foundation for improved understanding of *Brucella* infection mechanisms.

**Graphical Abstract:**

**Figure:**
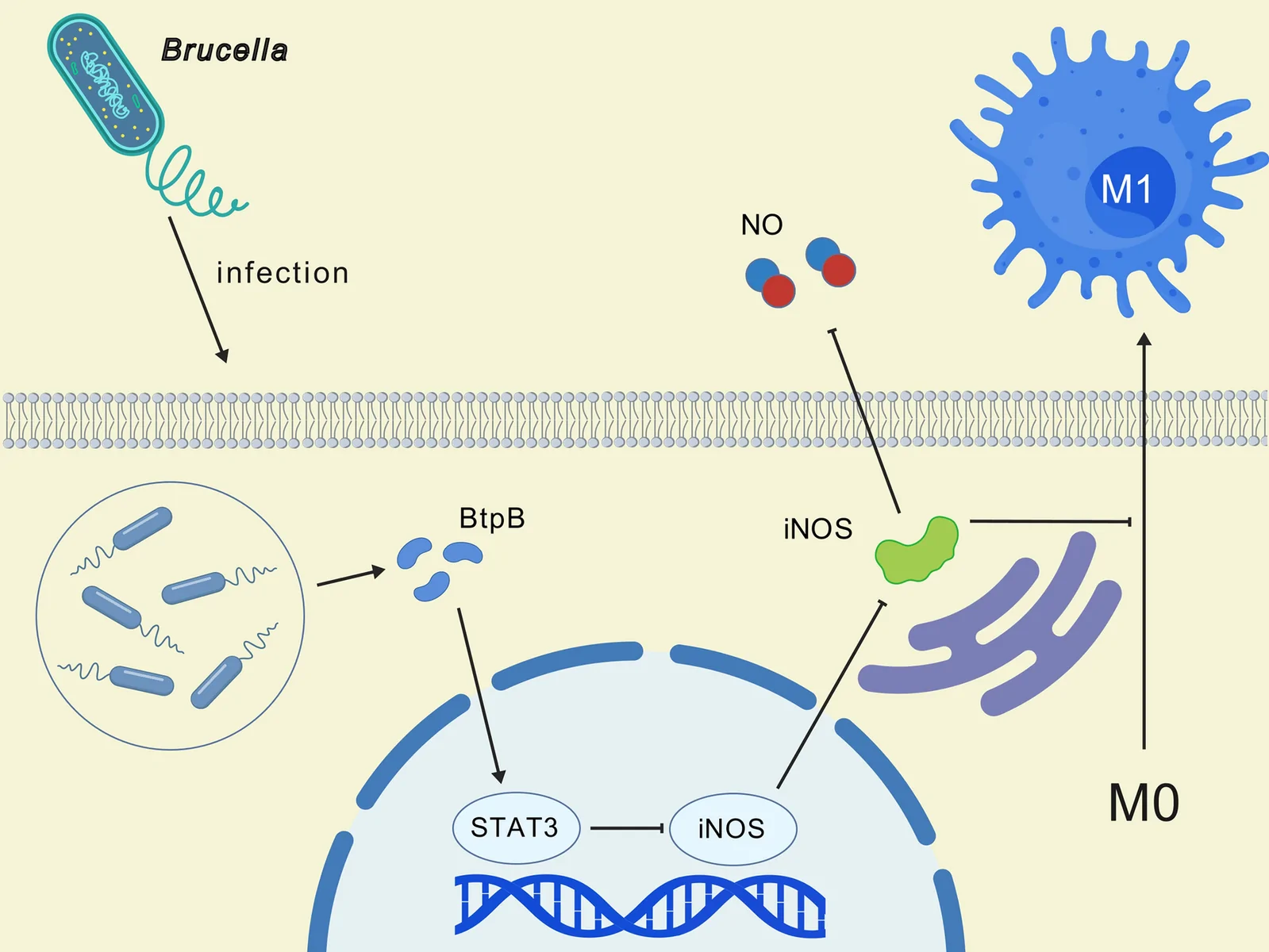

**Supplementary Information:**

The online version contains supplementary material available at https://doi.org/10.1186/s13567-026-01833-8.

## Introduction

*Brucella* is a Gram-negative, facultative intracellular pathogen that often appears as coccoid or short rods. It primarily causes brucellosis, which affects diverse domesticated mammals, including cattle, sheep, pigs, and dogs, as well as humans. The clinical manifestations of brucellosis in infected hosts include fever, arthritis, and reproductive dysfunction [1–3]. As an intracellular pathogen, *Brucella* employs numerous virulence factors to evade host adaptive and innate immune responses. Consequently, despite macrophages eliminating most *Brucella* cells following infection, a small fraction of the load evades the immune system, thereby enabling the pathogen to invade, survive, and proliferate within the host [4, 5]. This prolonged survival of the bacterium in host cells disrupts cellular signaling pathways and triggers complex host reactions [6].

Macrophages exhibit distinct phenotypes and functions in response to various microenvironmental signals, a process termed macrophage polarization [7]. Two distinct macrophage phenotypes have been identified: classically activated or inflammatory macrophages (M1-type) and alternatively activated macrophages (M2-type). M1 macrophages that are polarized via the classical activation pathway produce proinflammatory cytokines and drive robust inflammatory responses. However, these responses may also lead to tissue damage [8–10]. M1 macrophages are associated with pathogen clearance and antigen presentation to T lymphocytes to initiate adaptive immunity, which is reflected in the expression of costimulatory molecules linked to antigen presentation in these cells [11]. M2 macrophages that are polarized through the alternative activation pathway are generally considered to be anti-inflammatory. This cell type maintains metabolic homeostasis, repairs damaged tissues, and promotes T helper type 2 (Th2) immune responses. Four M2 subtypes (M2a, M2b, M2c, and M2d) have been defined depending on specific stimuli. These subtypes differ in surface markers, secreted cytokines, and biological functions [12, 13].

Signal transducer and activator of transcription 3 (STAT3) is a latent transcription factor that is activated transiently upon binding to polypeptide receptors at the cell surface. Activated STAT3 transmits transcription signals from cytokine and growth factor receptors on the plasma membrane to the nucleus [14]. As a critical hub for multiple signaling pathways, STAT3 plays a pivotal role in macrophage polarization. Key STAT3-associated pathways involved in macrophage polarization include the PI3K/AKT, Notch, NF-κB, Wnt, JAK/STAT, and mitogen-activated protein kinase (MAPK) signaling pathways, among others [15, 16]. STAT3 is a multifunctional protein that is regulated by diverse cytokines and growth factors. It influences metabolism, immune-inflammatory responses, and tissue repair via JAK/STAT signaling. Studies on the involvement of JAK/STAT3 in macrophage polarization revealed that both the inhibition and activation of this pathway can enhance M2 macrophage polarization in diverse disease contexts. Increased JAK2/STAT3 activity elevates cytokine levels required for M2 macrophage polarization and reduces the levels of proinflammatory factors, including interleukin 6 (IL-6), interferon gamma (IFN-γ), and tumor necrosis factor alpha (TNF-α), thereby mitigating pancreatic inflammation and injury in a severe acute pancreatitis rat model [17, 18]. In contrast, inhibiting the JAK/STAT3 pathway promotes M2 macrophage polarization, which was found to lower proinflammatory cytokines, upregulate the levels of anti-inflammatory factors, and attenuate tissue inflammation and damage in myocardial ischemia/reperfusion injury animal models [19].

The *Brucella* effector protein BtpB is a critical regulatory factor of the type IV secretion system (T4SS). BtpB suppresses the expression of Toll-like receptor 2 (TLR2) and Toll-like receptor 4 (TLR4), and attenuates activation of the NLRP3 inflammasome [20, 21]. We previously demonstrated that deletion of *btpB* (Δ*btpB*) increases the secretion of proinflammatory cytokines IL-1β, IL-6, and TNF-α in alveolar macrophages [21]. Additionally, BtpB promotes apoptosis, blocks autophagic flux, and inhibits autophagy during *Brucella* infection [22]. The type III secretion system effector SarA/SteE in *Salmonella* participates in the regulation of macrophage polarization, which influences the intracellular survival of this pathogen [23]. *Mycobacterium tuberculosis* infection activates the NOTCH signaling pathway to upregulate the expression of the gene for suppressor of cytokine signaling 3, thereby promoting M1 macrophage polarization [24]. As the Δ*btpB* mutant strain leads to heightened expression of proinflammatory cytokines in alveolar macrophages [21], we hypothesized that BtpB may similarly be implicated in macrophage polarization during *Brucella* infection. Therefore, we investigated the regulatory role of BtpB in *Brucella*-induced M1/M2 polarization of macrophages in vitro through immunofluorescence, reverse transcription quantitative PCR (RT-qPCR), western blot, and flow cytometry analyses. Furthermore, by employing STAT3 inhibition and overexpression approaches, we explored BtpB-mediated regulation of STAT3 to determine the functional role of STAT3 in BtpB-driven macrophage polarization. Thus, the study provides a foundation for further elucidation of the mechanisms that underpin chronic infection of macrophages by *Brucella*.

## Materials and methods

### Biosafety statement

All experiments were conducted in full compliance with the Biosafety Regulations for Pathogenic Microorganism Laboratories (Decree No. 424, 2004, promulgated by the State Council of the People’s Republic of China) and were approved by the Biosafety Committee of Northwest A&F University. All animal experiments adhered strictly to the Guidelines for Ethical Care and Use of Laboratory Animals and were reviewed and approved by the Institutional Animal Ethics and Use Committee.

### Bacteria, cells, and culture media

*Brucella suis* strain S2 was obtained from Shaanxi Provincial Veterinary Drug Control Institute and cultured in tryptic soy broth (TSB; Sigma-Aldrich, St. Louis, MO, USA) or tryptic soy agar (TSA; Sigma-Aldrich) at 37 °C. The BtpB mutant strain (Δ*btpB*) was constructed via homologous recombination, whereas the complemented strain (C-Δ*btpB*) was generated by electroporating the recombinant plasmid pBB-Amp-*BtpB* into *B. suis* Δ*btpB*, as previously described [21]. Murine macrophage RAW264.7 cells and relevant plasmids were maintained in our laboratory repository.

### *Brucella* infection

Macrophage RAW264.7 cells (1 × 10^6^ cells/well) were seeded in six-well plates and incubated overnight at 37 °C with 5% CO_2_, following which they were infected with strains *B. suis* S2, Δ*btpB*, or C-Δ*btpB* at a multiplicity of infection of 1:100. After 4 hours of infection, cells were washed 3 times with sterile phosphate buffer saline (PBS) to remove extracellular bacteria. Fresh Dulbecco's Modified Eagle Medium (DMEM; Solarbio Science & Technology Co. Ltd, Beijing, China) containing kanamycin (50 μg/mL; YuanYe Bio-Tech, Shanghai, China) was added and incubated for 1 h at 37 °C with 5% CO_2_, followed by three additional PBS washes. Cells were then replenished with DMEM containing kanamycin (25 μg/mL), with this time point designated as 0 h. Bacterial intracellular survival was assessed at 4, 12, and 24 h post-infection (hpi).

### Plasmid construction

The STAT3 expression plasmid was designed using the *STAT3* sequence (NM_009787) available in the National Center for Biotechnology Information (NCBI) databases. All specific primers were designed using Primer Premier software (PREMIER Biosoft, San Francisco, CA, USA) and were synthesized by Sangon Biotech (Shanghai, China). All primer sequences are detailed in Additional file 2. Target DNA fragment was amplified by PCR, separated by electrophoresis in a 1% agarose gel, and purified using a DNA gel extraction kit (TianGen, Beijing, China). The pEGFP-C1 vector was digested with restriction enzymes and ligated with the STAT3 fragment. The ligation product was transformed into *Escherichia coli* DH5α competent cells. Single colonies were selected, expanded, and subjected to plasmid extraction using an endotoxin-free plasmid miniprep kit (TianGen).

### Cell transfection

RAW264.7 cells were cultured to optimal confluency, then washed 3 times with PBS, followed by the addition of DMEM (2 mL); cells were then detached by gentle pipetting. The cell suspension was transferred to centrifuge tubes and centrifuged at 1000 rpm for 5 min. After cell counting, 1 × 10^6^ cells were seeded per well in six-well plates and cultured at 37 °C with 5% CO_2_ for 12–24 h. For transfection, Opti-MEM medium (200 μL) was aliquoted into sterile microtubes (Solarbio Science & Technology Co. Ltd.), followed by addition of plasmid DNA and FuGENE HD transfection reagent (Roche, Basel, Switzerland) at a 1 μg:3 μL ratio. The mixture was incubated at room temperature in the dark for 20 min, and then was added evenly to designated wells. The final transfection efficiency was examined by fluorescence microscopy and confirmed by western blotting; the overexpression effect is shown in Additional file 1.

### Reverse transcription quantitative PCR

Total RNA was extracted from cells using TRIzol reagent (Accurate Biology, Changsha, China) following the manufacturer’s protocol. RNA was reverse-transcribed into complementary DNA (cDNA) using a PrimeScript RT Master Mix kit (Vazyme, Nanjing, China). Quantitative PCR (qPCR) was performed on a LightCycler 480 System (Roche) with the SYBR Green Master Mix (Vazyme). Gene expression levels were calculated using the 2^−ΔΔCt^ method. All data are presented as mean ± standard error of the mean (SEM) of triplicate experiments.

### Western blot

Infected RAW264.7 cells were lysed in ice-cold RIPA buffer (Beyotime, Shanghai, China) for 30 min on ice. Lysates were centrifuged at 12 000 rpm for 15 min at 4 °C, and supernatants were collected for protein quantification using the BCA assay (Thermo Fisher Scientific, Waltham, MA, USA). Protein samples were mixed with 5× sodium dodecyl sulfate (SDS) loading buffer (Biosharp Life Sciences, Hefei, China), heated at 100 °C for 10 min, and separated by 10% SDS-polyacrylamide gel electrophoresis (PAGE). Proteins were transferred to PVDF membranes (Millipore, Merck KGaA, Burlington, MA, USA). Membranes were blocked with 5% skim milk (BD Biosciences, San Jose, CA, USA), in TBST (Tris-buffered saline with 0.1% Tween 20 detergent) for 2 h at room temperature, then incubated with anti-nitric oxide synthase (NOS2), anti-STAT3, or anti-β-actin primary antibodies (Abcam, Waltham, MA, USA) overnight at 4 °C. After five washes in TBST (5 min each), membranes were incubated with horseradish peroxidase (HRP)-conjugated secondary antibodies (Goat Anti-Rabbit IgG; Proteintech) for 1 h at room temperature. Signals were detected using ECL substrate (Bio-Rad Laboratories, Hercules, CA, USA) and visualized with an Imaging System (Bio-Rad Laboratories).

### Murine infection

Female BALB/c mice (6 weeks old) were purchased from Chengdu Dossy Experimental Animal Co., Ltd. (Chengdu, China) Mice were divided into four groups and injected intraperitoneally with 100 μL of sterile PBS or with PBS containing 1 × 10^6^ CFU *B. suis* S2, Δ*btpB*, or C-Δ*btpB* cells. At least three mice per group were euthanized via cervical dislocation under anesthesia at 7 and 14 dpi. Body weights were recorded and spleens were aseptically harvested for further analysis.

### Immunohistochemistry

Spleen tissues were fixed with 4% paraformaldehyde (MiShushengwu, Xi'an, Shaanxi, China), dehydrated through graded ethanol series, embedded in paraffin, and sectioned. The sections were then mounted on glass slides, deparaffinized, and rehydrated using an automated stainer (Leica Microsystems, Wetzlar, Germany). Antigen retrieval solution was added onto the glass slides (pH 6.0) at 95 °C for 20 min. The slides were incubated with anti-NOS2 rabbit polyclonal antibody (1:200 dilution; Abcam) at 4 °C overnight, followed by biotinylated goat anti-rabbit secondary antibody (1:500; Proteintech, Rosemont, IL, USA) at 37 °C for 30 min. Signal amplification was achieved using HRP-conjugated streptavidin (Proteintech) and visualized with DAB chromogen (MiShushengwu). Sections were counterstained with hematoxylin and imaged under a light microscope (Nikon Corp., Tokyo, Japan).

### Immunofluorescence assay

RAW264.7 cells (2 × 10^5^ cells/well) were seeded on glass coverslips in 24-well plates and cultured overnight at 37 °C with 5% CO_2_. Cells were infected as described above. At 4 and 24 hpi, cells were fixed with 4% paraformaldehyde for 15 min and then permeabilized with 0.2% Triton X-100 (Sigma-Aldrich) for 15 min at 37 °C. Primary anti-NOS2 antibody (1:200; Abcam) was applied overnight at 4 °C. Cells were washed and incubated with conjugated donkey anti-rabbit immunoglobulin G (IgG; 1:500; Proteintech) for 1 h at room temperature in the dark. Nuclei were stained with DAPI (1 μg/mL; MiShushengwu) for 10 min. Coverslips were mounted with antifade medium (MiShushengwu) and imaged using a confocal laser scanning microscope.

### Lactate and glucose metabolism assays

Lactate levels were quantified using an L-Lactic Acid (L-LA) Assay Kit (Beyotime) according to the manufacturer's protocol. Briefly, infected macrophages were homogenized in ice-cold extraction buffer (1 mL) and centrifuged at 12 000 rpm for 10 min at 4 °C. Supernatants were analyzed at 450 nm.

Glucose consumption was measured via a Glucose Assay Kit (Solarbio Science & Technology Co. Ltd.). Standards or samples (5 μL) were mixed with glucose assay reagent (185 μL), mixed on a vortex, and centrifuged at 5000 rpm for 2 min. Samples were heated at 95 °C for 8 min and then cooled to 4 °C. Each reaction (180 μL) was transferred to a 96-well plate while avoiding bubbles, and absorbance was read at 630 nm using a microplate reader (Tecan Group Ltd, Zürich, Switzerland).

### Flow cytometry

RAW264.7 cells (1 × 10^6^/well) in 12-well plates were infected as described above. At 24 hpi, cells were fixed with 4% paraformaldehyde for 15 min followed by staining with FITC-conjugated anti-CD86^+^ and APC-conjugated anti-CD206^+^ (Thermo Fisher Scientific) for 1 h at 4 °C. Samples were washed with PBS and then analyzed on a BD FACSCanto II flow cytometer (BD Biosciences).

### Mouse spleen detection

 The obtained mouse spleens were first ground gently to obtain spleen cell suspensions. Mouse splenic macrophages were then isolated from the cell suspensions using a mouse spleen macrophage isolation kit (YaJi Biological, New Taipei City, Taiwan). The obtained macrophage suspensions were fixed with 4% paraformaldehyde for 30 min. After fixation, Fc receptor blocking reagent was added to block Fc receptors and avoid interference. Following blocking, anti-mouse CD11b^+^, anti-mouse CD86^+^, and anti-mouse CD206^+^ flow cytometry antibodies (Thermo Fisher Scientific) were added to the samples and incubated at 4 °C for 30–40 min. After incubation, samples were analyzed on a BD FACSCanto II flow cytometer.

### CCK-8 assay for drug concentration screening

RAW264.7 cells (2 × 10^4^/well) in 96-well plates were treated in triplicate with the STAT3 inhibitor Stattic (Selleck, Houston, TX, USA) at 0.5, 1, 2, 5, or 10 μM. The cells were incubated at 37 °C with 5% CO_2_ in saturated humidity for 24 h, followed by the addition of CCK-8 reagent (10 μL; Merck) and a further 2-h incubation. Absorbance at 450 nm was measured using a microplate reader.

### Enzyme-linked immunosorbent assay

Cytokine levels were detected using enzyme-linked immunosorbent assay (ELISA) kits (Fankew, Shenzhen, China) according to the manufacturer’s instructions. In brief, cell culture supernatants (100 μL) were added to 96-well plates and incubated at 37 °C for 1 h. Samples were then washed 3 times with PBST, then biotinylated anti-IL-10 antibody (1:1000) was added (100 μL/well) and the incubation continued at 37 °C for 1 h. Samples were then washed 3 times with PBST and HRP-conjugated streptavidin (1:5000) was applied (100 μL/well) for 30 min at 37 °C. After final washes, TMB substrate (90 μL) was added and the samples were incubated in the dark for 15 min. Finally, 2 M H_2_SO_4_ (50 μL) was added and absorbance was measured at 450 nm using a microplate reader.

### Statistical analysis

All experiments were performed with at least three independent biological replicates. Statistical analyses were conducted using SPSS 26.0 software (IBM Corp., Armonk, NY, USA). Comparisons between two groups utilized two-tailed Student's t-tests, while one-way analysis of variance (ANOVA) with Tukey's post-hoc test was applied for multi-group comparisons. Significance thresholds were set at *p < 0.05, **p < 0.01, and ***p < 0.001. Graphical presentations were generated using GraphPad Prism 9.0 (GraphPad Software, San Diego, CA, USA), with values presented as mean ± SEM.

## Results

### BtpB suppresses M1 macrophage polarization in RAW264.7 macrophages

**Figure 1:**
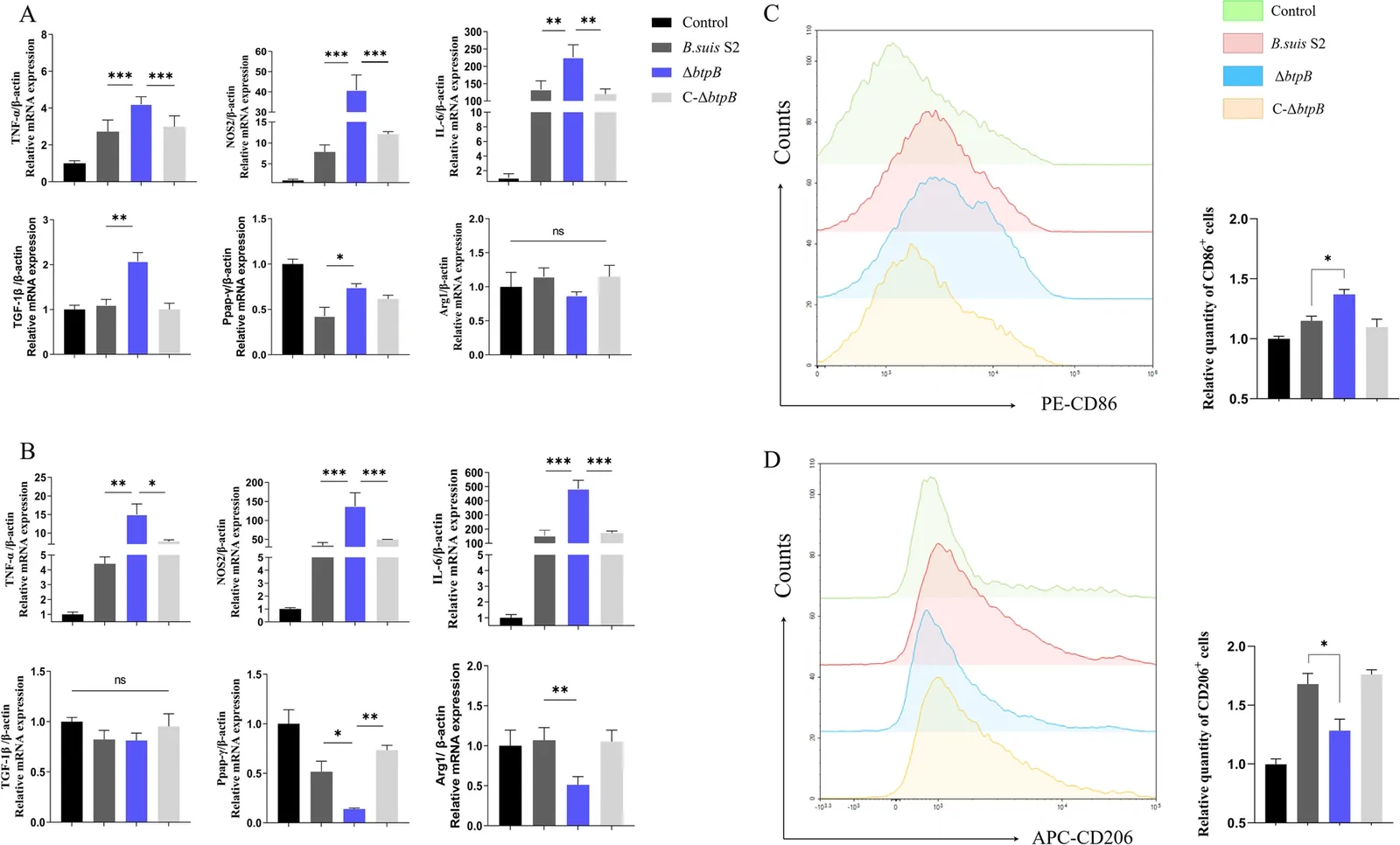
**The effect of BtpB on polarization of RAW264.7 macrophages.** **A** M1/M2 polarization factors at 4 hours post-infection as assessed by reverse transcription quantitative PCR (RT-qPCR). **B** M1/M2 polarization factors at 24 hpi assessed by RT-qPCR. **C**, **D** Detection of CD86 (M1 surface marker) and CD206 (M2 surface marker) expression in RAW264.7 cells at 24 hpi using flow cytometry. The experiments were performed in triplicate and the data are presented as the mean ± standard error of the mean. Asterisks denote a significant difference at *p < 0.05, **p < 0.01, and ***p < 0.001. Arg-1, Arginase-1; CD86, M1 surface marker; CD206, M2 surface marker; IL, interleukin; NOS2, nitric oxide synthase; TGF-1β, transforming growth factor beta 1; TNF-α, tumor necrosis factor alpha.

The role of BtpB in macrophage polarization was investigated first by examining changes in messenger RNA (mRNA) levels of polarization-related factors in RAW264.7 macrophages following infection with the *B. suis* S2, Δ*btpB*, or C-Δ*btpB* strain. The mRNA levels of the M1 polarization markers NOS2, IL-6, and TNF-α in cells infected with the BtpB deletion strain (Δ*btpB*) were significantly higher than those in cells infected with either the wild-type (WT; *B. suis* S2) or complemented strains (C-Δ*btpB*) at 4 hpi (Figure 1A). In parallel, the mRNAs of the M2 polarization markers transforming growth factor beta 1 (TGF-1β) and peroxisome proliferator–activated receptor γ (Ppar-γ) were also elevated in macrophages infected with the Δ*btpB* strain compared to cells infected with *B. suis* S2, whereas arginase-1 (Arg-1) expression was not significantly different between the WT, Δ*btpB*, and C-Δ*btpB* groups (Figure 1A). The levels of M1 polarization markers in Δ*btpB*-infected cells remained significantly higher than those in the *B. suis* S2 and C-Δ*btpB* groups at 24 hpi, whereas the M2 polarization markers *Ppar-γ* and *Arg-1* at this timepoint were lower in macrophages infected with the mutant strain compared to infection with either the WT or C-Δ*btpB* strains (Figure 1B).

The expression of the M1 surface marker CD86 and the M2 surface marker CD206 in RAW264.7 cells following *Brucella* infection was subsequently assessed by flow cytometry. RAW264.7 cells infected with the Δ*btpB* strain exhibited significantly higher levels of CD86 at 24 hpi (Figure 1C) than cells infected with the WT *B. suis* S2 strain, whereas CD206 expression was reduced markedly (Figure 1D). In summary, infection of macrophages with the *B. suis* mutant strain defective in *btpB* triggered M1 polarization, which suggests that BtpB suppresses M1 polarization but promotes M2 polarization of macrophages during *Brucella* infection.

### BtpB promotes M2 polarization of mouse splenic macrophages

We further validated the role of BtpB in macrophage polarization at the animal model level. By examining the levels of multiple M1 and M2 polarization-related cytokines in mouse serum at 1 and 2 weeks post-infection (wpi), we found that at 1 wpi, the Δ*btpB*-infected group showed significantly higher levels of TNF-α and iNOS than the *B. suis* S2- or C-Δ*btpB*-infected groups, whereas the levels of M2-related cytokines such as IL-10 and TGF-β1 were lower than those in the *B. suis* S2- or C-Δ*btpB*-infected groups. No differences were observed in IL-6 and Arg1 levels, which may be attributable to compensatory mechanisms through alternative pathways.

**Figure 2:**
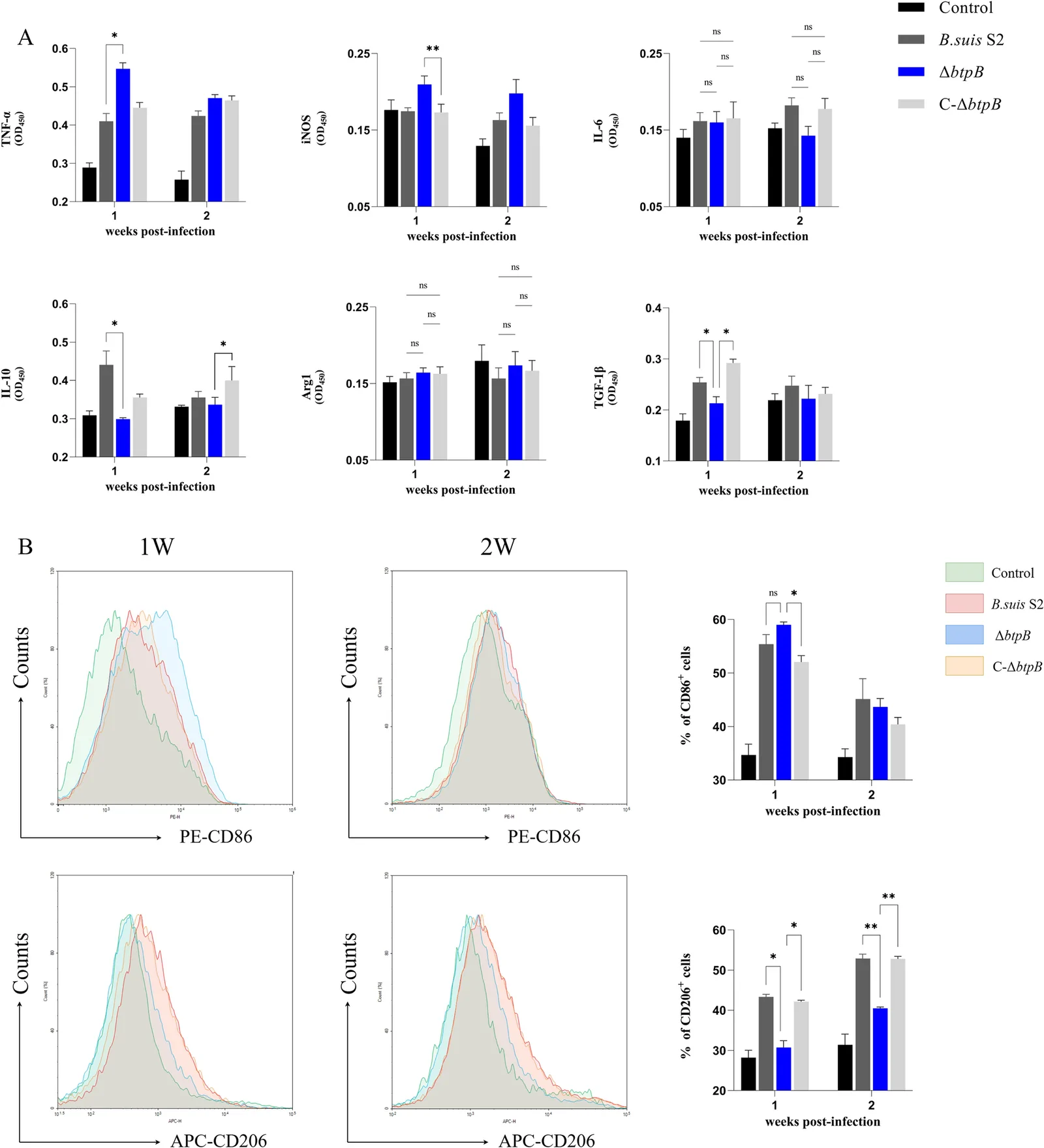
**BtpB promotes M2 polarization of mouse splenic macrophages. A** Levels of M1- and M2 polarization-related cytokines in mouse serum at 1 and 2 weeks post-infection (wpi) with the wild-type *B. suis* S2, the Δ*btpB* mutant, and complemented C-Δ*btpB* strains. **B** Polarization status of mouse splenic macrophages at 1 and 2 wpi. The experiments were performed in triplicate, and the data are presented as the mean ± standard error of the mean. Asterisks denote a significant difference at *p < 0.05, **p < 0.01, and ***p < 0.001. Arg-1, Arginase-1; CD86, M1 surface marker; CD206, M2 surface marker; IL, interleukin; iNOS, inducible nitric oxide synthase (NOS2); TGF-1β, transforming growth factor beta 1; TNF-α, tumor necrosis factor alpha.

Flow cytometric analysis of splenic macrophages collected at 1 and 2 wpi revealed that M2 polarization in the Δ*btpB*-infected group was significantly lower than that in both the *B. suis* S2 and C-Δ*btpB* groups during the first 2 weeks. Regarding M1 polarization levels, although no significant difference was detected between the Δ*btpB*-infected group and the *B. suis* S2-infected group, M1 polarization levels were significantly higher in the Δ*btpB* group than in the C-Δ*btpB* group, still demonstrating the role of BtpB in suppressing M1 polarization. These results demonstrate that BtpB continues to exhibit M1-suppressive and M2-promoting effects at the animal model level, corroborating our conclusions derived from cellular experiments (Figure 2).

### BtpB suppresses intracellular NOS2 expression during *Brucella* infection

**Figure 3:**
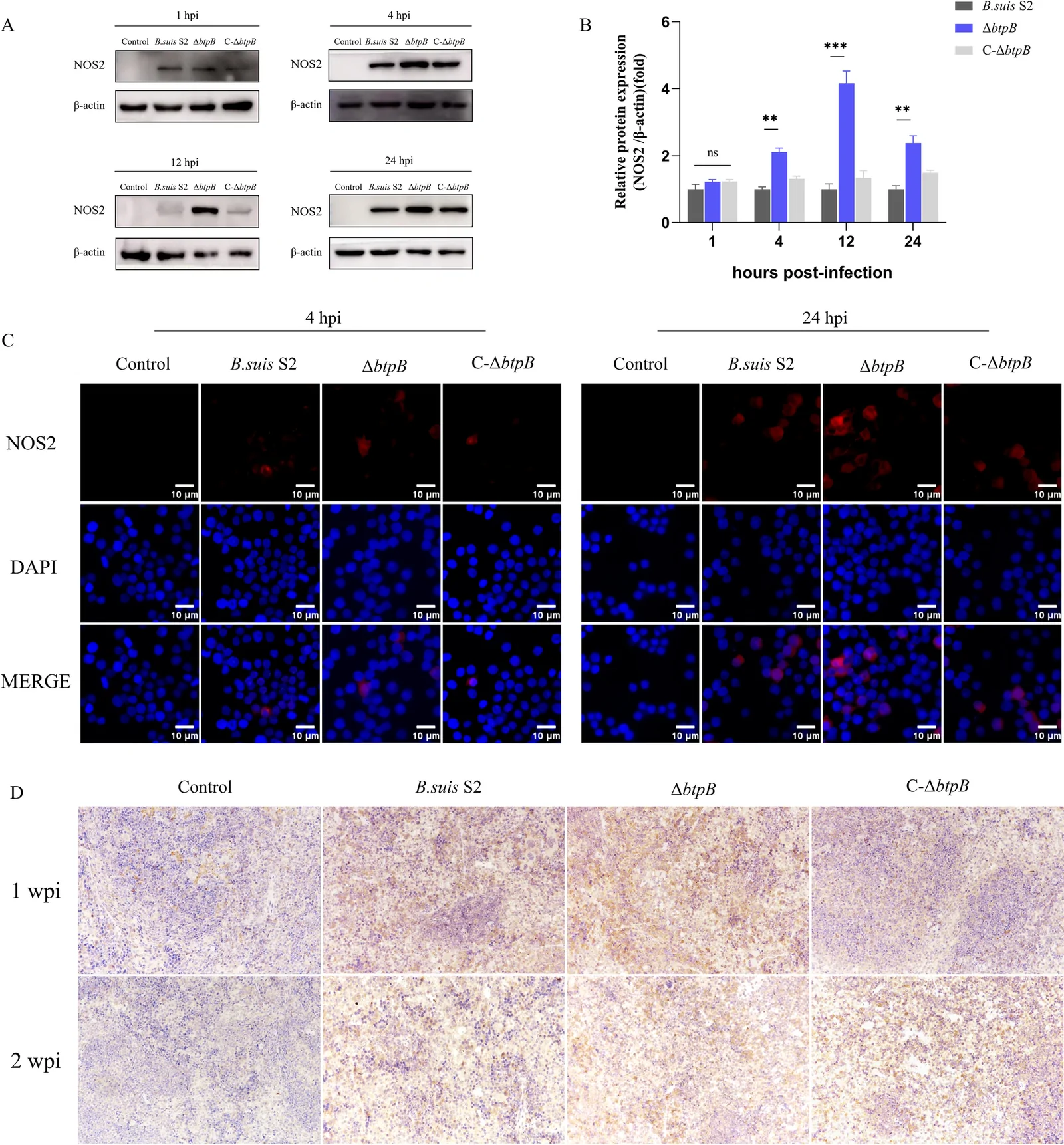
**BtpB suppresses NOS2 in the mouse spleen during* Brucella * infection.A**, **B** Expression of nitric oxide synthase (NOS2) detected by western blotting in RAW264.7 macrophages at 1, 4, 12, 24 hours post-infection (hpi). **C** Immunofluorescence staining for NOS2 (red) in RAW264.7 cells at 4 and 24 hpi with the wild-type *B. suis* S2, Δ*btpB* mutant, and complemented C-Δ*btpB* strains. **D** Immunohistochemistry observation of NOS2 expression in the spleen of BALB/c mice at 1 and 2 weeks post-infection (wpi). The experiments were performed in triplicate, and the data are presented as the mean ± standard error of the mean. Asterisks denote a significant difference at *p < 0.05, **p < 0.01, and ***p < 0.001. NOS2, Nitric oxide synthase.

To further validate the impact of BtpB on the polarization-related functional protein NOS2, we detected the expression levels of NOS2 protein in RAW264.7 cells at 1, 4, 12, 24 hpi. Macrophages infected with the Δ*btpB* mutant exhibited significantly higher NOS2 expression compared to cells infected with the*B. suis* S2 or C-Δ*btpB* strain at 4, 12, and 24 hpi, although not at 1 hpi (Figure 3A). The results of the immunofluorescence assay (IFA) further revealed that NOS2 expression in cells infected with the mutant strain was elevated markedly at both 4 and 24 hpi compared to other infections with the other strains (Figure 3C), a result that is consistent with the western blot and RT-qPCR results. These findings suggest that BtpB suppresses expression of the polarization-related protein NOS2 in *B. suis*-infected macrophages.

The gene for NOS2 is generally not expressed at all or is expressed at low levels under normal physiological conditions, but NOS2 is induced in macrophages when the host is exposed to external stimuli that trigger inflammatory responses; thus, NOS2 expression mirrors the inflammatory status of tissues [25]. Although the expression of NOS2 in spleens of mice infected with the *B. suis* S2, Δ*btpB*, or C-Δ*btpB* strain was elevated in all cases compared to spleens of uninfected mice 1 wpi (Figure 3D), the number of NOS2-positive cells increased markedly in spleens infected with the Δ*btpB* mutant strain compared to infection with either the WT *B. suis* S2 or C-Δ*btpB* strain. The NOS2-positive cell counts remained higher in the spleens of all infected mice compared with those in uninfected animals at 2 wpi, but no significant differences were observed. Thus, deletion of *btpB* would appear to upregulate the expression of NOS2 in the murine spleen during the early stage of infection, suggesting that BtpB exerts an inhibitory effect on *NOS2* expression during *Brucella* infection.

### BtpB enhances glucose uptake while reducing lactate expression during the early phase of infection

**Figure 4:**
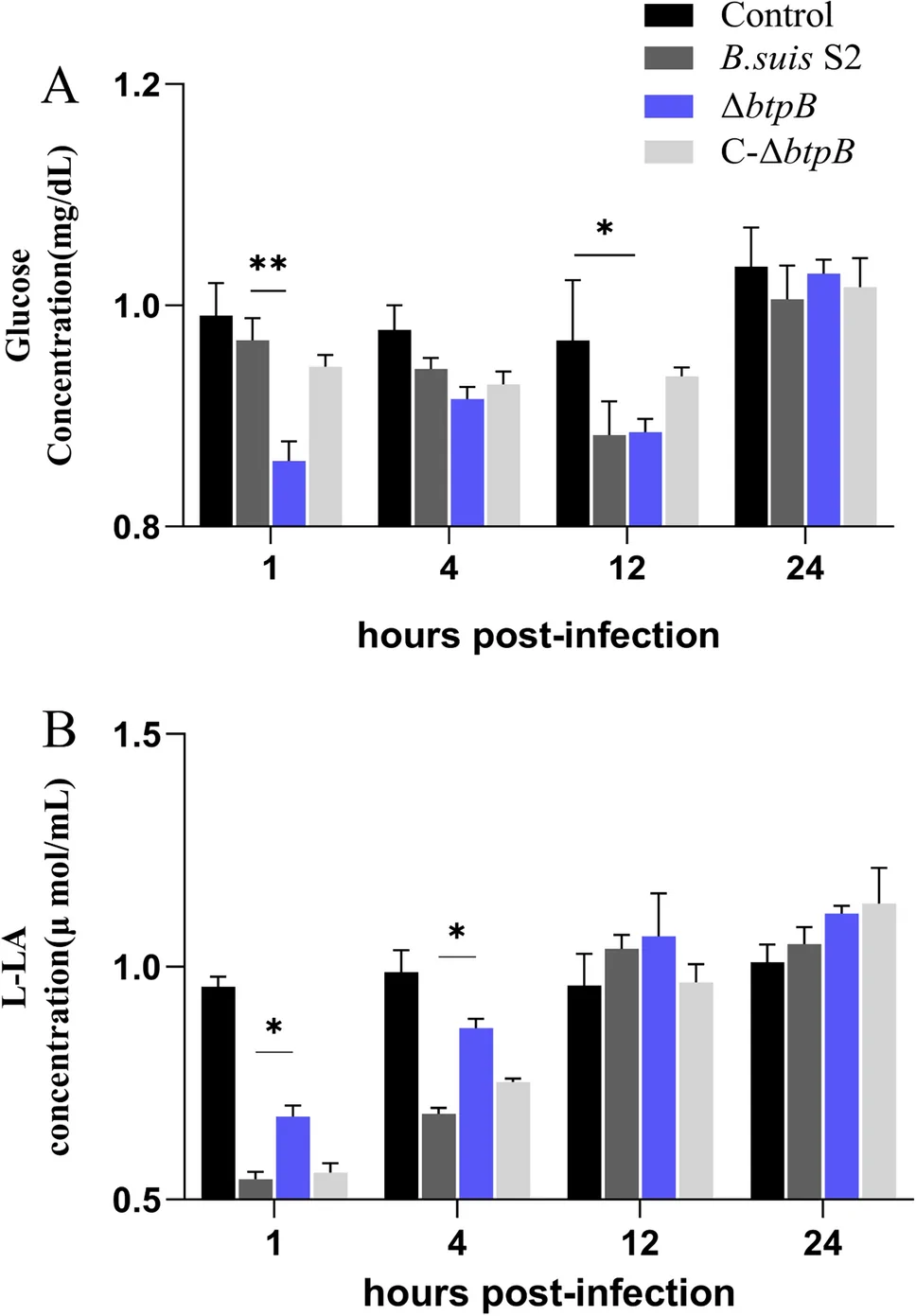
**Changes in cellular metabolism after** ***Brucella* infection**. **A** Detection of glucose consumption in cell supernatants of RAW264.7 macrophages at 1, 4, 12, and 24 hours post-infection (hpi) with the wild-type *B. suis* S2, Δ*btpB* mutant, and complemented C-Δ*btpB* strains. **B** Detection of lactic (L-LA) production in cell supernatants of RAW264.7 macrophages at 1, 4, 12, and 24 hpi after infection with the wild-type *B. suis* S2, Δ*btpB* mutant, and complemented C-Δ*btpB* strains. The experiments were performed in triplicate, and the data are represented as the mean ± standard error of the mean. Asterisks denote a significant difference at *p < 0.05, **p < 0.01, and ***p < 0.001.

M1 macrophages exhibit increased glucose uptake, enhanced glycolytic activity, and attenuated tricarboxylic acid cycle flux, whereas M2 macrophages demonstrate augmented oxidative phosphorylation [26]. Therefore, we quantified glucose consumption and lactate secretion in cell culture supernatants of RAW264.7 macrophages at 1, 4, 12, and 24 hpi. Glucose levels in the supernatant of cells infected with the Δ*btpB* mutant strain were reduced significantly compared to those in the supernatant of cells infected with the WT *B. suis* S2 strain at 1 hpi, whereas no significant differences were observed at 4, 12, or 24 hpi (Figure 4A). Additionally, Δ*btpB*-infected cells exhibited significantly higher lactate secretion than cells infected with the WT strain at 1 and 4 hpi, but no significant differences were detected at 12 and 24 hpi (Figure 4B). These findings collectively demonstrate that BtpB suppresses M1 macrophage polarization during the early infection phase with *B. suis*, thereby regulating glucose metabolism and lactate expression in host cells.

### BtpB upregulates STAT3 expression and facilitates phospo-STAT3 nuclear translocation during *Brucella* infection

**Figure 5:**
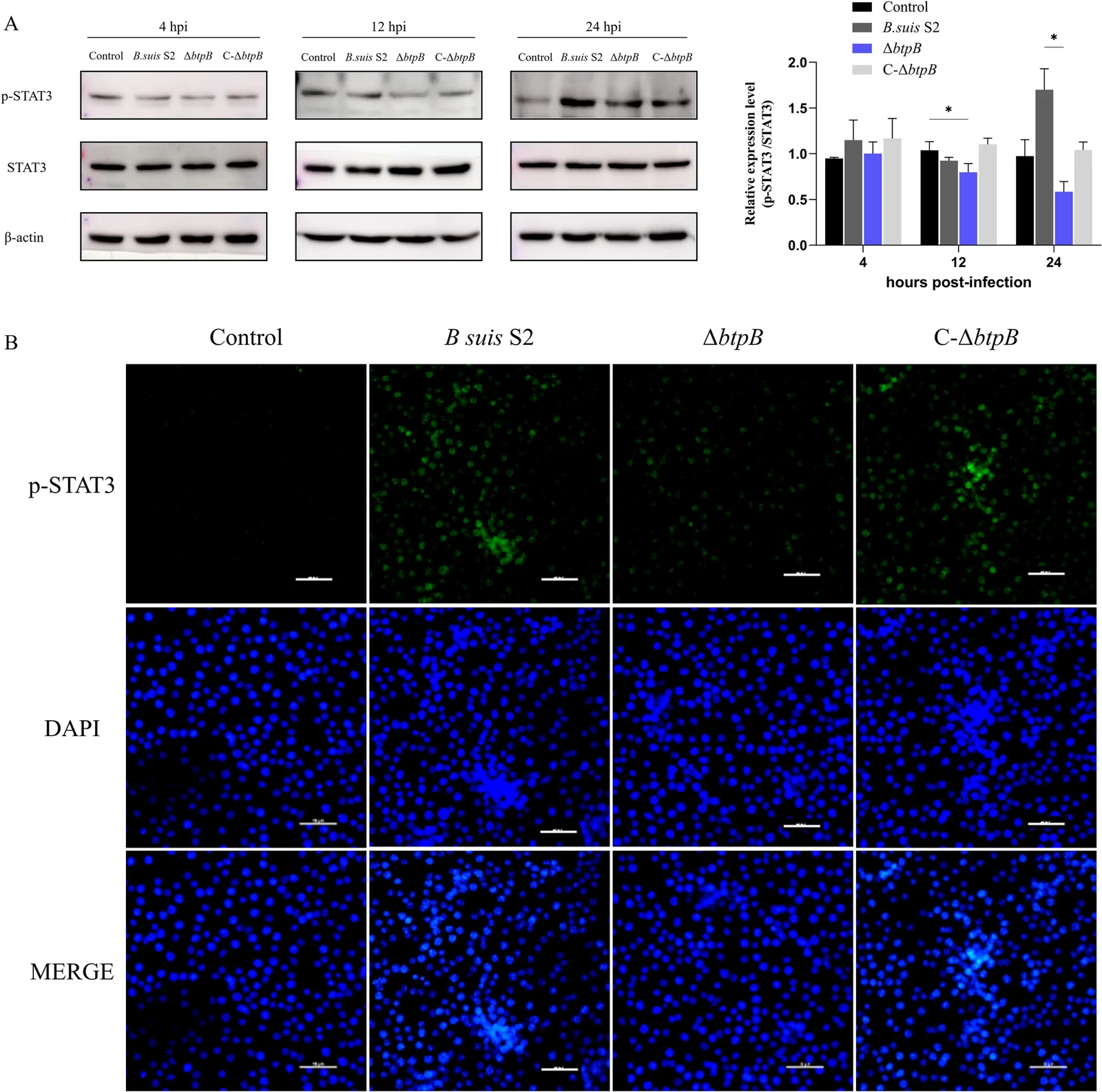
**BtpB modulates the level of p-STAT3 in RAW264.7 macrophages during** ***Brucella* infection.** **A** Expression of p-STAT3 and STAT3 in RAW264.7 cells detected by western blotting at 4, 12, and 24 hours post-infection (hpi) with the wild-type *B. suis* S2, Δ*btpB* mutant, and complemented C-Δ*btpB* strains. **B** BtpB promotes p-STAT3 nuclear transfer in RAW264.7 cells detected by immunofluorescence assay (IFA) at 24 hpi with *B. suis* S2, Δ*btpB*, and C-Δ*btpB* strains. The experiments were performed in triplicate, and the data are presented as the mean ± standard error of the mean. Asterisks denote a significant difference at *p < 0.05, **p < 0.01, and ***p < 0.001. (p)-STAT3, (Phospho)-signal transducer and activator of transcription 3.

Prior studies have established the involvement of STAT3 in macrophage polarization processes [27]. The potential regulatory role of BtpB on STAT3 signaling in host cells was investigated by analyzing both total STAT3 and phospho-STAT3 (p-STAT3) expression in macrophages at 4, 12, and 24 hpi after infection with the *B. suis* S2, Δ*btpB*, or C-Δ*btpB* strain. No significant differences were observed at 4 or 12 hpi, but cells infected with the deletion strain (Δ*btpB*) exhibited a marked reduction of phosphorylated (p)-STAT3/STAT3 at 24 hpi compared to cells infected with the WT *B. suis* S2 strain (Figure 5A).

IFA of infected RAW264.7 macrophages at 24 hpi was performed to assess further the nuclear translocation dynamics of p-STAT3. Consistent with the western blot results, The IFA revealed that cells infected with Δ*btpB* exhibited significantly diminished nuclear accumulation of p-STAT3 compared to either *B. suis* S2- or C-Δ*btpB*-infected cells (Figure 5B). Thus, BtpB upregulates STAT3 expression and facilitates the nuclear translocation of activated p-STAT3 during macrophage infection by the WT *B. suis* S2 strain.

### BtpB inhibits macrophage M1 polarization through STAT3 activation

**Figure 6:**
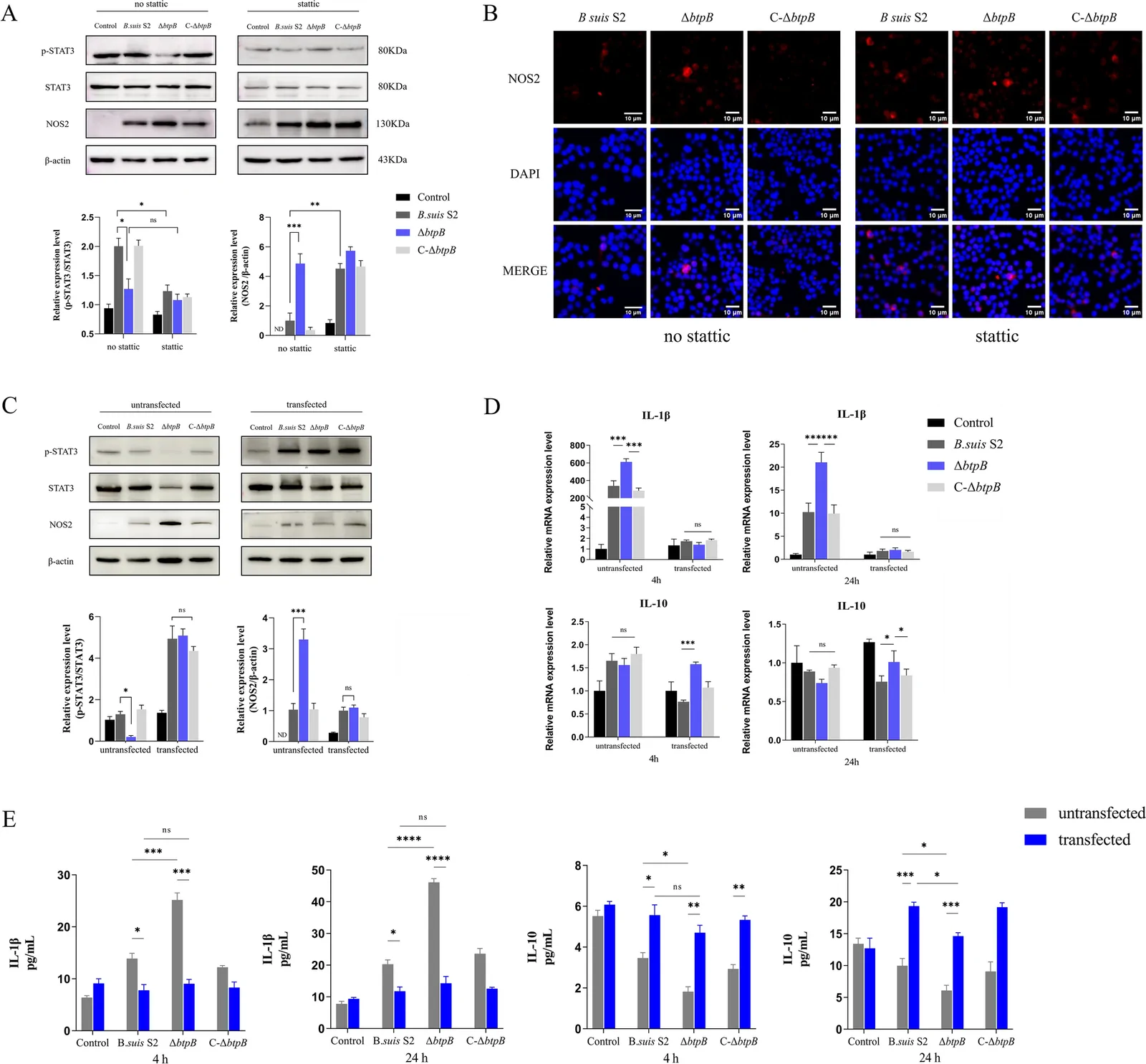
**BtpB inhibits macrophage M1 polarization through STAT3-mediated signaling. A** Expression of NOS2 detected by western blotting in RAW264.7 cells treated with Stattic (5 μM) after infection with the wild-type *B. suis* S2, Δ*btpB* mutant, or complemented C-Δ*btpB* strain. **B** Expression of NOS2 detected by immunofluorescence assay in RAW264.7 cells treated with Stattic (5 μM) after infection with *B. suis* S2, Δ*btpB*, or C-Δ*btpB*. **C** Expression of NOS2 detected by western blotting in RAW264.7 cells overexpressing *STAT3* after infection with *B. suis* S2, Δ*btpB*, or C-Δ*btpB*. **D** Effect of overexpression of *STAT3* on intracellular polarization factors IL-1β and IL-10 detected by quantitative PCR in RAW264.7 cells after infection with *B. suis* S2, Δ*btpB*, or C-Δ*btpB*. **E** Detection of IL-1β and IL-10 expression in supernatants after overexpression of *STAT3* in the enzyme-linked immunosorbent assay (ELISA) . The experiments were performed in triplicate, and the data are represented as the mean ± standard error of the mean. Asterisks denote a significant difference at *p < 0.05, **p < 0.01, and ***p < 0.001. IL, Interleukin; NOS2, nitric oxide synthase; (p)-STAT3, (phospho)-signal transducer and activator of transcription 3.

Signal transducer and activator of transcription 3 functions as a pivotal hub that integrates multiple signaling pathways and plays a critical role in macrophage polarization. Therefore, the contribution of BtpB-mediated regulation of STAT3 to macrophage polarization was investigated further in RAW264.7 cells that were pretreated with the specific STAT3 inhibitor Stattic followed by *Brucella* infection. Protein lysates were collected at 24 hpi for western blot analysis to assess NOS2 expression. In the absence of Stattic treatment, macrophages infected with the Δ*btpB* mutant strain showed significantly reduced STAT3 expression and dramatically elevated NOS2 levels compared to cells infected with either the WT *B. suis* S2 or complemented C-Δ*btpB* strain (Figure 6A). In contrast, Stattic-treated macrophages exhibited no significant differences in NOS2 levels following infection with *B. suis* S2, Δ*btpB*, or C-Δ*btpB*. Moreover, cells infected with the WT *B. suis* S2 or C-Δ*btpB* strain displayed significantly increased NOS2 expression in the Stattic-treated condition compared to untreated conditions (Figure 6A). The detection of NOS2 by the IFA corroborated the western blot findings (Figure 6B). These results indicate that inhibition of STAT3 activity promotes NOS2 expression in *Brucella*-infected macrophages.

RAW264.7 cells were transfected with the pEGFP-C1-STAT3 overexpression plasmid followed by *Brucella* infection to compare the effect of STAT3 overexpression on NOS2 levels in transfected and untransfected macrophages. In non-transfected cells, Δ*btpB* infection significantly upregulated NOS2 protein levels compared to infection with the WT or C-Δ*btpB* strains (Figure 6C). In transfected cells, no significant differences in NOS2 expression were observed among the three sets of infected cells, although Δ*btpB*-infected macrophages exhibited markedly reduced NOS2 levels in the transfected group compared to the non-transfected counterparts (Figure 6C). These results demonstrate that STAT3 overexpression suppresses *Brucella*-induced NOS2 expression in macrophages.

The impact of STAT3 on macrophage polarization was examined by assessing mRNA levels of polarization cytokines IL-10 and IL-1β in STAT-overexpressing RAW264.7 macrophages at 4 and 24 hpi following infection with the WT *B. suis* S2, Δ*btpB* mutant, or complemented C-Δ*btpB* strain. The expression of IL-1β was reduced significantly in STAT3-transfected cells compared to non-transfected controls at 4 hpi following infection, with Δ*btpB*-infected transfected cells exhibiting the lowest IL-1β levels. No significant intergroup differences were observed in transfected cells at 24 hpi, although all transfected macrophages showed lower IL-1β expression compared to the non-transfected counterparts (Figure 6D). Conversely, Δ*btpB* infection induced significantly higher IL-10 mRNA levels in transfected cells at both 4 and 24 hpi compared to infection with *B. suis* S2 or C-Δ*btpB* (Figure 6D). ELISA quantification of IL-1β and IL-10 proteins in cell supernatants corroborated these findings (Figure 6E). Thus, BtpB modulates STAT3 expression to regulate the expression of macrophage polarization-associated cytokines, thereby inhibiting M1 polarization and enhancing M2 polarization.

### STAT3 modulates the intracellular proliferation of *Brucella*

**Figure 7:**
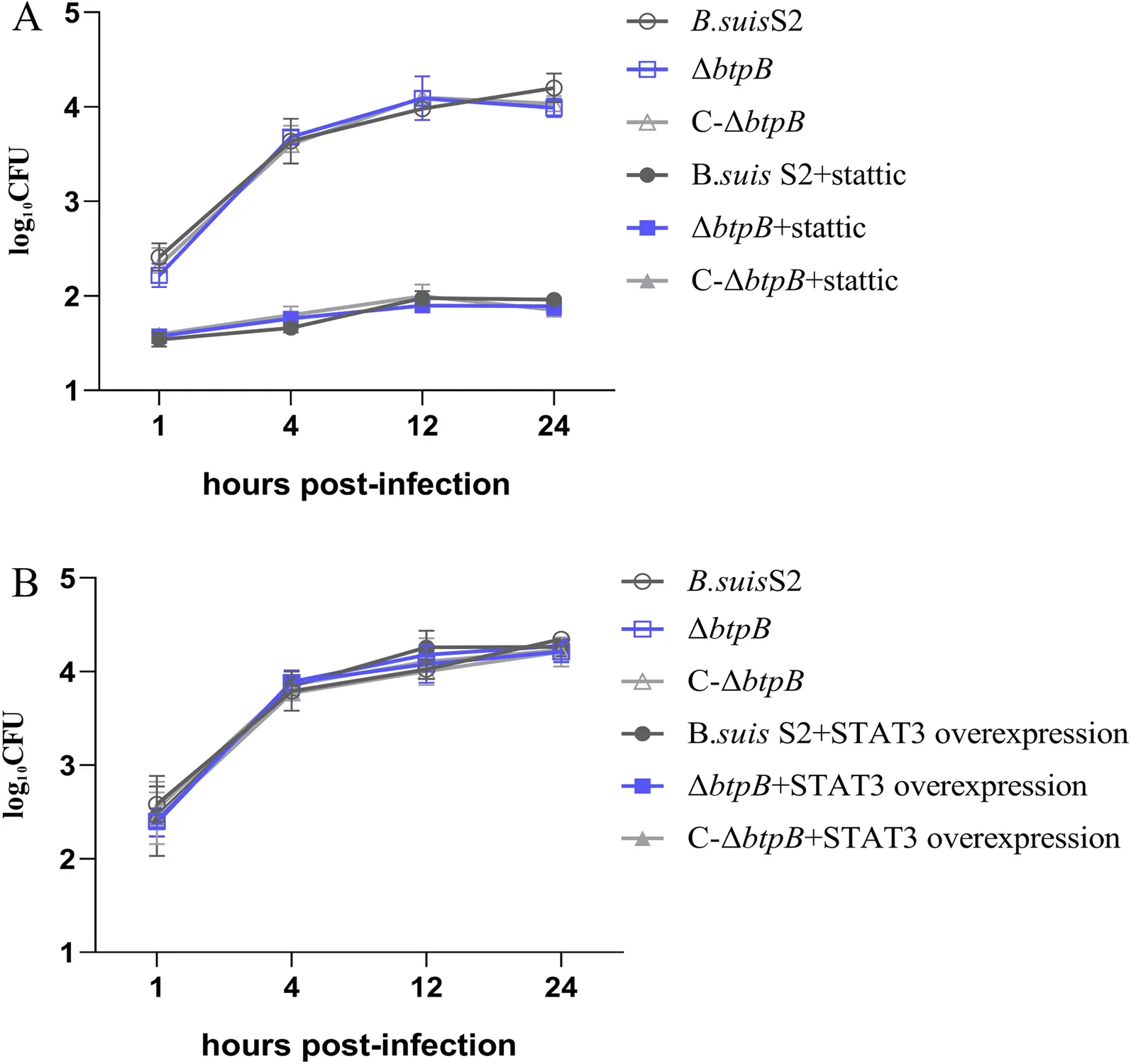
**The impact of STAT3 on intracellular proliferation of *Brucella.*** **A** Intracellular proliferation of the wild-type *B. suis* S2, Δ*btpB* mutant, and complemented C-Δ*btpB* strain in RAW264.7 cells treated with Stattic (5 μM). **B** Intracellular proliferation of *B. suis* S2, Δ*btpB*, or C-Δ*btpB* in RAW264.7 cells overexpressing STAT3. The experiments were performed in triplicate, and the data are presented as mean ± standard error of the mean. Asterisks denote a significant difference at *p < 0.05, **p < 0.01, and ***p < 0.001.

The impact of STAT3 on the intracellular proliferation of *Brucella* was investigated in RAW264.7 macrophages that were pretreated with Stattic or transfected with the pEGFP-C1-STAT3 overexpression plasmid, followed by infection with the WT *B. suis* S2, Δ*btpB* mutant, or complemented C-Δ*btpB* strain. Intracellular bacterial loads were enumerated at 1, 4, 12, and 24 hpi. STAT3 inhibition significantly reduced intracellular replication (Figure 7A), although no significant intergroup differences were observed between macrophages infected with *B. suis* S2, Δ*btpB*, or C-Δ*btpB*. In contrast, STAT3 overexpression did not alter the intracellular survival of these strains with no significant differences compared to non-transfected controls (Figure 7B). These findings demonstrate that suppression of STAT3 activity impairs *Brucella* intracellular survival, whereas STAT3 overexpression exerts no detectable effect on *Brucella* proliferation.

## Discussion

BtpB is a critical effector protein of the Type IV secretion system (T4SS) in *Brucella* and harbors a Toll/IL-1 receptor/Resistance protein (TIR) domain that potently suppresses the TLR2, TLR4, and TLR9 signaling pathways [21]. BtpB mutant strains of Brucella induce significant upregulation of major histocompatibility complex (MHC)-II molecules in antigen-presenting cells [20], which is a phenomenon that potentially drives macrophage polarization. In the present study, *Brucella* infection assays in macrophages and murine infection experiments were performed using the previously established Δ*btpB* mutant and complemented C-Δ*btpB* strains. Infection with the Δ*btpB* mutant promoted the upregulation and downregulation of M1 and M2 polarization factors, respectively, while simultaneously inducing an increase in the expression of the polarization marker NOS2. Detection of glucose consumption and lactate secretion levels confirmed that deletion of *btpB* induces a metabolic shift toward M1 polarization in macrophages. These results collectively demonstrate that BtpB functions to inhibit M1 polarization while promoting M2 polarization. STAT3 plays a pivotal regulatory role in macrophage polarization. In our study, examination of the effect of BtpB on STAT3 in macrophages revealed that infection with the Δ*btpB* strain induced a significant reduction in STAT3 expression. We systematically assessed the effects of both STAT3 inhibition and overexpression on macrophage polarization to determine further whether BtpB-mediated regulation of STAT3 contributes to the promotion of M2 macrophage polarization. This analysis demonstrated that BtpB suppresses M1 polarization of macrophages through STAT3 expression modulation. Moreover, investigation of the impact of STAT3 on *Brucella* intracellular proliferation indicated that STAT3 inhibition significantly impairs *Brucella* replication.

KEGG-enriched pathways primarily converge on NLR signaling transduction, antigen processing and presentation, and TLR signaling pathways during early and intermediate stages of M1 polarization [28]. In a previous study, we demonstrated that BtpB suppresses TLR2 and TLR4 expression in macrophages while attenuating NLRP3 inflammasome activation, thereby impairing early host immune responses [21]. In the present study, we therefore extended our investigation to the effects of BtpB on macrophage polarization. Analysis of mRNA levels for multiple M1/M2 polarization-associated cytokines revealed that deletion of *btpB* triggered elevated concentrations of M1-polarizing factors (Figure 1A, 1B). Although M2-polarizing factors showed downregulated expression at 24 hpi, these factors exhibited transient upregulation comparable to that of M1-associated factors during early infection (4 hpi; Figure 1B). This paradoxical elevation presumably reflects compensatory anti-inflammatory responses by the host that counteracts Δ*btpB*-induced hyperinflammatory signaling. However, this upregulation was transient, ultimately progressing toward an M1 polarization-dominant phenotype. We speculate that the robust M1 polarization response triggered during the early stage of Δ*btpB* infection may activate the host's compensatory anti-inflammatory mechanism. Specifically, high levels of proinflammatory cytokines (such as TNF-α, IL-1β, and IL-6) may directly induce the expression of SOCS3, a negative regulator of STAT3, thereby forming a negative feedback loop [29]. Concurrently, excessive activation of the IL-6/STAT3 signaling axis may induce the secretion of anti-inflammatory factors such as IL-10, serving to suppress excessive inflammatory damage. Furthermore, previous studies have reported that the enhanced glycolysis associated with M1 polarization may lead to lactate accumulation, and lactate can promote the expression of M2-related genes such as Arg1 through histone lactylation modification, driving the phenotypic transition of macrophages from proinflammatory M1 to anti-inflammatory M2 [30, 31]. Finally, the enhanced level of the M1-polarization surface marker CD86 coupled with downregulated CD206 expression conclusively demonstrates the inhibitory effects of BtpB on M1 polarization (Figures 1C, D, 2B).

NOS2 is induced in M1-polarized macrophages upon activation to generate nitric oxide, which in turn mediates critical antimicrobial functions, including the direct cytocidal activity against intracellular pathogens, elimination of host cell-invading microbes, and activation of adaptive immunity through Th1 cell polarization and antigen presentation enhancement [10, 29]. Infection of macrophages with the Δ*btpB* mutant induced sustained upregulation of NOS2 protein expression with statistical significance across multiple timepoints, which demonstrates BtpB-mediated suppression of NOS2. These findings also indicate that Δ*btpB*-driven NOS2 upregulation may potentiate nitric oxide-dependent antimicrobial activity, thereby triggering a heightened proinflammatory immune response in the host [32].

Macrophage polarization is linked intricately to alterations in cellular metabolism. M1 polarization is characterized by enhanced glycolysis, pentose phosphate pathway activation, and fatty acid synthesis, which manifests as escalated glucose consumption and intensified glycolytic efficacy. Conversely, M2 polarization preferentially utilizes oxidative phosphorylation and fatty acid oxidation [31, 33, 43]. During polarization, M1 macrophages undergo metabolic reprogramming that is characterized by accelerated glycolytic flux. This bioenergetic shift rapidly generates ATP to meet the high-energy demands of inflammatory responses [34]. The glycolytic ATP surge fuels the robust expression and release of proinflammatory mediators, including iNOS, IL-1β, and IL-6. Additionally, glycolytic metabolites such as lactate accumulate extracellularly, which actively modulates inflammatory cascades [35]. Our previous studies have shown that BtpB exhibits NAD^+^ hydrolase activity, which reduces intracellular NAD^+^/NADH ratios during *Brucella* infection of macrophages [34, 36]. The pivotal role of NAD^+^ in coordinating glycolysis and oxidative phosphorylation [35, 37, 44] may provide a mechanistic explanation for the observed metabolic reprogramming in Δ*btpB*-infected cells that is characterized by an increase in glucose uptake and elevated lactate secretion (Figure 4). The underlying mechanism may involve BtpB-mediated hydrolysis of NAD^+^ that results in depletion of intracellular NAD^+^, which in turn subsequently triggers disruption of glycolytic flux, thereby impairing macrophage M1 polarization. This cascade may explain why metabolic alterations predominantly occur during early infection stages and supports the inhibitory effect of BtpB on M1 polarization.

The alteration in p-STAT3/STAT3 levels in macrophages following *Brucella* infection confirmed our hypothesis that BtpB activates the expression and phosphorylation of STAT3 in host cells (Figure 3). STAT3 inhibition in *B. suis* S2-treated macrophages resulted in the significant upregulation of NOS2 expression, which demonstrated the suppressive effect of STAT3 on NOS2. Conversely, STAT3 inhibition exhibited no significant impact on STAT3 expression levels in cells infected with the Δ*btpB* mutant, and NOS2 levels consequently remained unaltered under STAT3-suppressed conditions (Figure 6B). This observation suggests that the compensatory upregulation of STAT3 triggered by the *btpB* deletion counteracts Stattic-induced reduction in STAT3 expression, thereby maintaining homeostatic NOS2 levels. Following STAT3 overexpression, intracellular NOS2 levels in the macrophages infected with the Δ*btpB* mutant were reduced markedly compared to the levels in the non-transfected controls, which indicates that STAT3 overexpression functionally compensates for STAT3 inhibition mediated by Δ*btpB*. Consequently, elevated NOS2 expression in Δ*btpB*-infected cells without transfection of the STAT3 expression plasmid was normalized to levels comparable to cells infected with WT *B. suis* upon STAT3 overexpression (Figure 6C). This compensation mechanism strongly supports BtpB-mediated STAT3 regulation as a critical pathway for suppressing M1 macrophage polarization during *Brucella* infection. Parallel quantification of polarization-associated cytokines reinforced this conclusion: markedly higher IL-1β levels were observed in cells infected with the Δ*btpB* mutant compared to cells infected with the WT strain in the absence of STAT3 overexpression. This result was reversed dramatically in cells that overexpressed STAT3, with Δ*btpB*-infected cells displaying significantly lower IL-1β levels than macrophages infected with the WT *B. suis* S2 strain (Figure 6D, 6E). Conversely, IL-10 expression followed an inverse pattern. These data provide validation that BtpB modulates macrophage polarization through STAT3-mediated regulatory networks, thereby establishing a crucial role for the BtpB-STAT3 axis in modulating host immune responses.

Numerous intracellular pathogens antagonize antimicrobial immunity by translocating effector proteins that suppress proinflammatory signaling [30, 38]. The effector protein SteE in *Salmonella* converts host intracellular serine/threonine kinase GSK3 into a tyrosine kinase, thereby activating STAT3 to drive macrophage M2 polarization, which is a state characterized by high M2 macrophage abundance that facilitates persistent bacterial infection [23, 39]. This phenomenon has also  been documented in *Trichinella spiralis* and *Mycobacterium tuberculosis* infections [24, 40]. Unlike the *Salmonella* T3SS effector SteE and *Mycobacterium tuberculosis* cell wall components (such as PIM2 and PPE-18), BtpB is translocated into host cells via the *Brucella* T4SS. Its unique secretion mechanism and subcellular localization likely determine the specific mode by which it regulates STAT3. Additionally, BtpB possesses the capacity to modulate Toll-like receptor signaling and dendritic cell activation, and this multi-target regulatory capability further underscores the complexity of *Brucella*'s immune evasion strategy. Inhibiting STAT3 activity reduced *Brucella* intracellular proliferation. However, our results showed that the presence or absence of BtpB did not alter bacterial survival within host cells (Figure 7). STAT3 inhibition may simultaneously affect multiple host antimicrobial mechanisms, with effects extending beyond the regulatory scope of the BtpB–STAT3 axis. For example, as a critical node in multiple cytokines signaling pathways, comprehensive inhibition of STAT3 may cause drastic alterations in the overall immune status of host cells, thereby masking the specific regulatory differences conferred by BtpB. As one of dozens of effector proteins in the *Brucella* T4SS, the impact of BtpB on intracellular bacterial proliferation through STAT3 modulation is likely compensated by other effector proteins (such as BtpA, SepA, VceC, etc.) or virulence factors, thereby maintaining the pathogen's replication capacity within host cells. Although STAT3 inhibition attenuates the bacterium’s capacity for persistent infection, the downregulation of STAT3 expression in cells infected with the Δ*btpB* mutant was insufficient to cause further reduction in intracellular survival compared to infection with *B. suis* S2. The underlying mechanisms warrant further investigation.

In summary, this study demonstrates that the *Brucella* effector protein BtpB suppresses macrophage M1 polarization and promotes M2 polarization by upregulating STAT3 expression. Concurrently, inhibition of STAT3 activity significantly attenuates *Brucella* intracellular proliferation within macrophages. These findings elucidate a critical mechanism by which BtpB modulates inflammatory responses during *Brucella*–host interactions. Given the pivotal role of M1 polarization in antibacterial defense, particularly in activating T lymphocytes and initiating adaptive immunity [11, 41, 42, 45], the M1-inhibitory property of BtpB unveils a novel avenue for developing live-attenuated *Brucella* vaccines.

## Supplementary Information

**Additional file 1. Validation of inhibitor treatment and overexpression.** (A) CCK8 screening for Stattic concentration. (B) Inhibitory effect of Stattic on STAT3 detected by qPCR. (C) PCR amplification of stat3 gene. (D) Fluorescence detection of pEGFP-C1-STAT3 vector transfection efficiency. (E) Western blot analysis to verify the effects of inhibition and overexpression.

**Additional file 2. Primers used in this study.**

## Data Availability

No datasets were generated or analyzed during the current study.
