# Supplementary material for: The Brucella effector protein BtpB facilitates STAT3 activation to regulate polarization in macrophages during infection
Source: Vet Res. 2026 Sep 2;57:163. doi: 10.1186/s13567-026-01833-8 (PMC13540847; doi:10.1186/s13567-026-01833-8)
Supplement: Supplementary file 1 [file 13567_2026_1833_MOESM2_ESM.doc]

| Class | Primer | Sequence（5’-3’） | Product size | NCBI Reference Sequence |
| --- | --- | --- | --- | --- |
| PCR | STAT3-F | TCGAGCTCAAGCTTCGAATTCATGGCTCAGTGGAACC | 2313bp | NM_213659 |
|  | STAT3-R | TTATCTAGATCCGGTGGATCCTCACATGGGGGAGGTA |  |  |
| RT-qPCR | TNF-α F  TNF-α R | GGGGATACATCCATCAGGGGT  GCTCGGACAGTCACTCACC | 163bp | [NM_011609.4](https://www.ncbi.nlm.nih.gov/entrez/viewer.fcgi?db=nucleotide&id=158533987) |
|  | IL-6 F  IL-6 R | CTGCAAGAGACTTCCATCCAG  AGTGGTATAGACAGGTCTGTTGG | 131bp | [NM_031168.2](https://www.ncbi.nlm.nih.gov/entrez/viewer.fcgi?db=nucleotide&id=930945753) |
|  | IL-10 F  IL-10 R | CTTACTGACTGGCATGAGGATCA  GCAGCTCTAGGAGCATGTGG | 101bp | [NM_010548.2](https://www.ncbi.nlm.nih.gov/entrez/viewer.fcgi?db=nucleotide&id=291575143) |
|  | IL-1β F | TCAAAGCAATGTGCTGGTGC | 133bp | [NM_008361.4](https://www.ncbi.nlm.nih.gov/entrez/viewer.fcgi?db=nucleotide&id=921274059) |
|  | IL-1β R | ACCTAGCTGTCAACGTGTGG |  |  |
|  | NOS2 F  NOS2 R | GTTCTCAGCCCAACAATACAAGA  GTGGACGGGTCGATGTCAC | 127bp | [NM_010927.4](https://www.ncbi.nlm.nih.gov/entrez/viewer.fcgi?db=nucleotide&id=927028884) |
|  | TGF-β F  TGF-β R | CCACCTGCAAGACCATCGAC  CTGGCGAGCCTTAGTTTGGAC | 91bp | NM_011577.2 |
|  | Ppar-γ F  Ppar-γ R | GGAAGACCACTCGCATTCCTT  GTAATCAGCAACCATTGGGTCA | 121bp | [NR_176971.1](https://www.ncbi.nlm.nih.gov/entrez/viewer.fcgi?db=nucleotide&id=2280970882) |
|  | Arg-1 F  Arg-1 R | CTCCAAGCCAAAGTCCTTAGAG  GGAGCTGTCATTAGGGACATCA | 184bp | [NM_007482.3](https://www.ncbi.nlm.nih.gov/entrez/viewer.fcgi?db=nucleotide&id=158966684) |
|  | β-actin F | TGGGTATGGAATCCTGTGGC | 200bp | [NM_007393.5](https://www.ncbi.nlm.nih.gov/entrez/viewer.fcgi?db=nucleotide&id=930945786) |
|  | β-actin R | GGAGCAATGATCTTGATCTTC |  |  |

**Additional file 2. Primers in this study**
